# Clinical Applications of Omics Technologies on ZHENG Differentiation Research in Traditional Chinese Medicine

**DOI:** 10.1155/2013/989618

**Published:** 2013-06-18

**Authors:** Ya-Nan Song, Gui-Biao Zhang, Yong-Yu Zhang, Shi-Bing Su

**Affiliations:** ^1^Research Center for Traditional Chinese Medicine Complexity System, Shanghai University of Traditional Chinese Medicine, 1200 Cailun Road, Pudong, Shanghai 201203, China; ^2^Research Center for Traditional Chinese Medicine and Systems Biology, Shanghai University of Traditional Chinese Medicine, 1200 Cailun Road, Pudong, Shanghai 201203, China

## Abstract

Traditional Chinese medicine (TCM) ZHENG is the basic concept of TCM theory. The effectiveness of TCM treatment depends on the accuracy of ZHENG differentiation. ZHENG differentiation, using the “four diagnostic methods,” has the drawbacks of subjectivity and variability. Following development of omics technologies, which study the functional activities of human body from a system-wide perspective, it has been more and more applied in study of objectivity differentiating TCM ZHENG and understanding its biological mechanisms. This paper reviewed the literatures of clinical TCM ZHENG differentiation researches, underlying omics technologies, and indicated the increased trends of related articles with four kinds of omics technologies, including genomics, transcriptomics, proteomics and metabolomics, and the correlations between ZHENG differentiation and findings in omics studies. Moreover, the paper summarized the typical omics application in common studied diseases and TCM ZHENGs and discussed the main problems and countermeasure of ZHENG differentiation researches. The work here may provide a reference for further research of TCM ZHENG differentiation using omics technologies.

## 1. Introduction

Traditional Chinese medicine (TCM) is a healthcare system with rich experience over 3000 years of continuous practice and refinement [1]. In several Asian countries, TCM acts as a pivot in servicing human healthcare, and in most Western countries, it is considered as a complementary or alternative medicine [2]. The effectiveness of TCM depends on the accurate ZHENG differentiation and treatment procedures (known as “Bian ZHENG Lun Zhi”) [3]. ZHENG, which is also called TCM syndrome or TCM pattern, is a profile of disharmonious symptoms and signs. TCM ZHENG is identified from a comprehensive analysis of clinical phenotypes based on the “four diagnostic methods,” namely, inspection, listening and smelling, inquiring, and palpation [4]. ZHENG differentiation, which is used to guide TCM treatment by acupuncture, herbal formulae, and so on, is one of the most important procedures in the practice of TCM. Different ZHENGs need different treatment strategies. Thus, the accuracy of ZHENG differentiation will directly influence the effectiveness of treatment.

ZHENG differentiation is not only important but also complicated in TCM. The ZHENG differentiation mainly relies on the experiential verdict of TCM practitioners, through symptoms, tongue appearance, and pulse feelings of patients, which is often considered to be subjective. So there is inevitable variability in different practitioners, even when the same patient was diagnosed [5]. On the other hand, the medical service based on TCM ZHENG differentiation may be difficult to be understood in the west. Therefore, in the era of complementary and alternative medicine, TCM ZHENG has encountered a hard nut to crack from biomedical science in the world due to a shortage of evidence-based theoretical interpretations and solid proof of ZHENG-based effect [6]. So it is inevitable to explore the biological basis of TCM ZHENG differentiation.

Omics represent the rigorous research of various collections of molecules, biological processes, or physiologic functions and structures as systems [7]. Omics including genomics, transcriptomics, proteomics, and metabolomics, have recently been widely used in the TCM ZHENG differentiation studies [8–11], with the rapid growth of large-scale detection technologies [12]. With the help of computational approaches, omics technologies focus on understanding functional activities from a systemswide perspective [13]. It is similar with the characteristics of TCM ZHENG, which pays much attention to integrity and dynamics. Different omics data helps to explore the variation of DNA, mRNA, protein, and metabolite in human body, and computational approaches help to explore molecular functional networks [14]. Academic researchers have already begun to focus on developing basic informatics tools that can integrate large quantities of omics data to mimic regulatory networks and cell function [15]. With the integration of high-throughput global genomic, transcriptomic, proteomic, metabolomic technologies, and huge datasets, omics technologies have become powerful tools for improving our knowledge of health and disease [16, 17].

In this study, the literatures on clinical TCM ZHENG differentiation research by omics technologies were screened, and the articles that applied omics technologies in TCM researches were reviewed. We hope our work could provide a reference for further TCM ZHENG differentiation studies by omics approaches.

## 2. A Literature Analysis for the Clinical TCM ZHENG Differentiation Research by Omics Technologies

The concept of genomics has been proposed for more than twenty years, but that of metabolomics was just proposed in 1999. Considering few clinical research studies of TCM ZHENG differentiation using metabolomics can be found; the data before 2003 were ignored in this review. Therefore, clinical studies of TCM ZHENG by omics technologies were searched in the past ten years. Most related research studies were found from the English-language database PubMed (http://www.ncbi.nlm.nih.gov/PubMed/) and the Chinese-language database CNKI (China National Knowledge Infrastructure, http://epub.cnki.net/). During the PubMed database search, “TCM ZHENG or TCM pattern or TCM syndrome” and “omics or genomics or transcriptomics or proteomics or metabolomics or systems biology” were limited within the scope of title and abstract. In order to find more articles, we add “gene or protein or metabolite” within the scope of title and abstract. After 524 unrelated studies were filtered out artificially, whose TCM ZHENG differentiation researches were without omics technologies, 90 articles were acquired from 2003 to October 31, 2012. Similarly, during the CNKI database search, “*证候*” (TCM ZHENG) and “*组学*” (omics) or “*基因* *组学*” (genomics) or “*转* *录* *组学*” (transcriptomics) or “*蛋白质* *组学*” (proteomics) or “*代* *谢* *组学*” (metabolomics) or “*系统生物学*” (systems biology) or “*基因* (gene)” or “*蛋白质* (protein)” or “*代谢物* (metabolite)” were limited within the scope of title and abstract. After 767 unrelated studies were filtered out, 140 articles were found from 2003 to October 31, 2012.

### 2.1. Overall Literature Profiles of Clinical TCM ZHENG Differentiation Research by Omics Technologies

**Figure 1:**
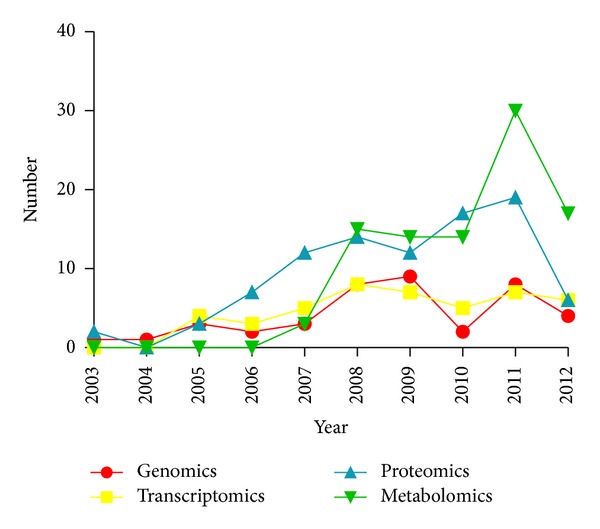
The trends of articles about clinical TCM ZHENG differentiation research with four omics technologies, including genomics, transcriptomics, proteomics, and metabolomics. The data were obtained from the PubMed database and the CNKI database in the past ten years (from 2003 to October 31, 2012).

Figure 1 shows the changing trend of clinical application of four omics technologies (genomics, transcriptomics, proteomics, and metabolomics) on TCM ZHENG differentiation research in the past ten years. Because there was a delay in the literature publication and collection process, the 2012 dataset was collected until October 31, 2012. So, not all of the data in 2012 was included in the annual statistics in this study. Except the year of 2012, Figure 1 shows an annual increase in number of publications. The number of articles has increased rapidly in the past ten years, which indicated an increasing attention on TCM ZHENG differentiation using omics technologies in the world. On the other hand, the proportion of studies using proteomics and metabolomics was rising over time, especially after 2006. While the number of studies using genomics and transcriptomics remained a small proportion of the total, suggesting that the technologies of genomics and transcriptomics had a lack of application on TCM ZHENG differentiation research.

### 2.2. Basic Diseases and ZHENGs Analysis in Clinical TCM ZHENG Differentiation Research by Omics Technologies

Currently, ZHENG differentiation is trying to combine with a biomedical diagnosis in TCM clinical practice. Therefore, among the studies of ZHENG differentiation, some involved diagnosis of diseases, while others did not involve.

**Figure 2:**
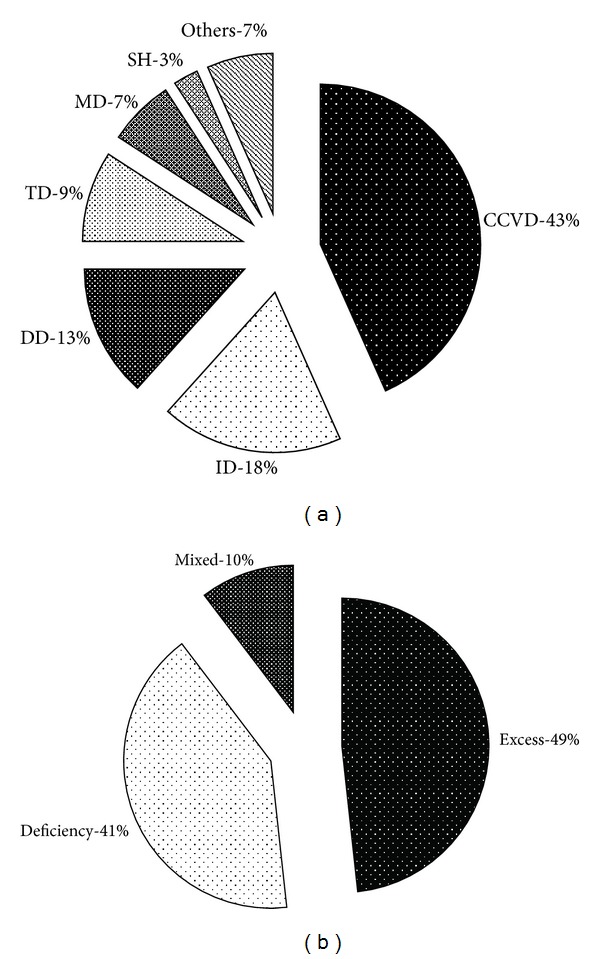
Basic diseases and ZHENGs analysis in clinical TCM ZHENG differentiation research by omics technologies. (a) The proportion of disease categories in clinical TCM ZHENG differentiation research. CCVD, cardiac-cerebral vascular disease; ID, immunological disease; DD, digestive diseases; TD, tumor; MD, metabolic disorders; SH, subhealth and others. (c) The proportion of TCM ZHENG categories in clinical TCM ZHENG differentiation research.

By limiting TCM ZHENG literatures to clinical studies, we counted the amount of studies related to different diseases. Then the diseases involved in these studies were classified into several categories, including cardiac-cerebral vascular disease (CCVD), immunological disease (ID), digestive diseases (DD), tumor (TD), metabolic disorders (MD), and subhealth (SH). The proportion of disease categories is shown in Figure 2(a). Moreover, the several most common diseases in TCM ZHENG differentiation studies by omics technologies are summarized in Table 1. In addition, the related literatures about these diseases are also listed in Table 1, which are all research articles included in PubMed. Figure 2(a) and Table 1 concluded the literature numbers of most diseases involved in modern TCM ZHENG differentiation studies. As shown in Table 1, the most researched diseases were coronary heart disease (10.43%), chronic liver disease (6.52%), hypertension (5.22%), chronic kidney disease (4.35%), hyperlipidemia and atherosclerosis (3.48%), chronic stomach disease (3.48%) and diabetes mellitus (3.48%), respectively. It not only suggested that the tendency of the researches using omics technologies was focused on chronic diseases, but also referred more biological evidence to right ZHENG differentiation and get better efficacy on these chronic diseases by TCM therapy. This point was the same with the previous ZHENG-related literature analysis [6].

Similarly, by limiting TCM ZHENG literatures to clinical studies, we counted the amount of studies related to different TCM ZHENG. Then the ZHENGs involved in these studies were classified into three categories, excess ZHENG, deficiency ZHENG, and mixed ZHENG. The proportion of ZHENG categories is shown in Figure 2(b). Moreover, the several most common ZHENGs in TCM ZHENG differentiation studies by omics technologies are summarized in Table 2. The related literatures about these ZHENGs are also listed in Table 2, which are all research articles included in PubMed. As shown in Figure 2(b), the categories of Excess ZHENG (48.3%) and deficiency ZHENG (41.4%) were studied in the similar proportion, while the category of mixed ZHENG was only studied in a small proportion (10.3%) due to the complexity of mixed ZHENG, which is excess and deficiency combination. On the other hand, most of TCM ZHENGs in Table 2 are excess ZHENGs. It suggested that the amount of excess ZHENG studied was larger than that of deficiency ZHENG or mixed ZHENGs studied. Since TCM theory, deficiency ZHENG or mixed ZHENG is much more complex than excess ZHENG, which usually appears in later stage of diseases. It implied that clinical TCM ZHENG studies using omics technologies only were the beginning. Except excess and deficiency ZHENG differentiation, there are cold and heat, exterior and interior, Yin and Yang, and so forth, in ZHENG differentiation. Different ZHENG differentiation guides to different treatment in TCM clinical practice.

### 2.3. Correlation Analysis between TCM ZHENG Differentiation and Findings in Omics Researches

In this review, we mainly searched the articles in clinical researches of TCM ZHENG differentiation using omics technologies from PubMed database. Among these researches, 47 articles have focused on mechanism of ZHENG, and 26 articles have focused on diagnosis and stratification of ZHENG. All these studies aim to differentiate ZHENG more scientifically and achieve higher efficacies for the treatment of disease. 

With the help of omics technologies, some key genes, proteins or metabolites were found, which may play an important role in the occurrence and development of TCM ZHENG; thus, the mechanism and the essence of ZHENG could be understood better. For example, previous genomics studies have found the relationships between interleukin-10 genotype and deficiency ZHENG in hepatitis B cirrhosis [29, 30] and between apolipoprotein polymorphisms and ZHENGs in type 2 diabetes mellitus [55]. Using transcriptomics, Ma et al. [22] have indicated that interleukin-8 might be related to the pathobiology of blood stasis ZHENG in cornary heart, and Chen et al. [64] have concluded that inflammatory-gene expression (ALOX5AP, S100A8 and S100A12) might be related to Cold ZHENG in hemodialysis. Proteomic and metabolomic technologies have been also applied to study mechanism of ZHENG. For instance, Zhao et al. [24] have observed that actin was associated with Qi deficiency and blood stasis ZHENG in unstable angina. Jian et al. [27] have found that arachidonic acid, octadecanoic acid, lactic acid, urea, and so forth are associated with heart-blood stasis ZHENG in coronary heart.

In ZHENG differentiation research of diagnosis and stratification of ZHENG, metabolomic technology was often used, while genomics was seldom used. The reason may be that metabolomics explores terminal product which is easy for diagnosis of TCM ZHENG. It also provides an evidence for TCM practitioners to diagnose patients from their appearance, such as tongues, faces. Using metabolomics, Qi-Yin deficiency was differentiated in type 2 diabetes patients [59], Cold and Heat ZHENGs were differentiated in rheumatoid arthritis patients [68], and so on. Moreover, transcriptomics and proteomics were also used to study diagnosis and stratification of ZHENG [34–36].

The correlation analysis indicated that omics technologies are promising tools for ZHENG differentiation researches. Though previous studies provided little data as the statistical strength of correlations between ZHENG differentiation and findings in omics studies at present, following the development of omics technologies, these technologies will be used to find more biomarkers for diagnosis of ZHENGs and explain the mechanism of ZHENGs.

## 3. Applications of Omics Technologies on Clinical Research of TCM ZHENG Differentiation

With the improvement of omics technologies, scientists have gradually realized that these omics technology-based diagnostic principles can be used as a bridge between TCM and conventional medicine [75]. At present, though the applications of omics technologies on TCM are much less popular than on conventional medicine, clinical TCM ZHENG differentiation studies by omics technologies have also achieved some great successes. There have been researchers applying omics technologies in the determination of TCM ZHENG to provide evidence for TCM by comparing the differences of DNA, RNA, proteins, and metabolites and eventually to disclose the material foundation and mechanism of TCM ZHENG. Here, we talk about the concepts and applications of omics technologies, including genomics, transcriptomics, proteomics, and metabolomics. Clinical TCM ZHENG differentiation was mostly researched in those diseases listed in Supplementary Table 1 available online at http://dx.doi.org/10.1155/2013/989618, and the most popularly studied TCM ZHENGs are listed in Supplementary Table 2.

### 3.1. Genomics

Genome refers to all of genetic material in a living organism, a virus or an organelle. Genomics, also known as gene polymorphism, is a tool for analyzing the correlation between genotypes and phenotypes of health or disease. It aims to detect the linear chromosomal sequence of model samples, as well as sequence differences between individuals. Annotating the genome, including defining coding and regulatory sequences, is also part of genomics [76]. The technologies include polymerase chain reaction-restriction fragment length polymorphism (PCR-RFLP), PCR-ligase detection reaction (PCR-LDR), single nucleoside polymorphisms (SNPs) microarray, and gene sequencing.

As shown in Tables 1 and 2 and Supplementary Tables 1-2, genomic technology plays an important role in the clinical TCM ZHENG differentiation researches in many diseases. In the field of coronary heart disease, it was found that the relationships really exist between blood-stasis ZHENG and the polymorphism of platelet activation genes [18] and angiotensin converting enzyme genes [21] and the relationships between phlegm-stasis ZHENG and the polymorphism of apolipoprotein E (ApoE) genes [19, 20]. In hepatitis B-caused cirrhosis (HBC), the studies suggested that interleukin-10-819C/T locus (TC plus CC genotype) is probably a risk factor in the occurrence of deficiency ZHENG [29], allele C in interleukin-10-819 locus may be related to spleen deficiency with overabundance of dampness ZHENG, and TT genotype in interleukin-10-819 locus may be related to liver stagnation ZHENG [30]. In hypertension, the relationships between some angiotensin-related genes and dual deficiency of Yin and Yang ZHENG, Yin deficiency and Yang hyperactivity ZHENG, phlegm-wetness ZHENG, and so forth has been studied [39, 40]. In chronic kidney disease, the substantial genetic basis for excess and deficiency ZHENG was searched from NPHS1 gene and NPHS2 gene polymorphisms [46] and megsin gene polymorphism [47]. In hyperlipidemia, it was found that polymorphism of ApoE gene is related in a certain degree to liver-kidney Yin deficiency ZHENG, spleen-kidney Yang deficiency ZHENG, Qi-stagnation-caused blood-stasis ZHENG, and so forth [50]. In diabetes mellitus, ApoE gene polymorphism was also explored, and it has proved that the Qi-Yin deficiency ZHENG in type 2 diabetes mellitus patients with macroangiopathy is associated with the ApoE E4 and E3 genotypes [55].

Moreover, Zhou et al. and Ding et al. have detected the linkage disequilibrium (LD) SNPs to explore the genetic traits of kidney-Yang deficiency ZHENG and found that kidney-Yang deficiency ZHENG is involved in special LD SNPs in the intragenic level and proved doublecortin domain containing 5 and other genes surrounding these SNPs display some relationships with key symptoms of kidney-Yang deficiency ZHENG [77, 78].

### 3.2. Transcriptomics

The TCM ZHENG differentiation has close intrinsic relations with not only the gene polymorphisms but also the difference of gene expression which is the research missions of transcriptomics. Transcriptomics, the next level down of genomics, deals with a transcript, that is, mRNA. Whereas the defining technology in genome sequencing is the automated DNA sequencer, in transcriptomics it is microarray hybridization or mRNA sequencing, which is detecting the level of mRNA in cells or tissues or the relative level between two states [76].

The applications of transcriptomics on TCM ZHENG researches are shown in Tables 1 and 2 and Supplementary Tables 1-2. In the coronary heart disease research, using gene chips technology, the correlation of inflammatory- and immune-related genes with blood-stasis ZHENG in coronary heart patients was revealed, and it was found that the hereditary correlated differential genes of blood-stasis ZHENG are closely associated with inflammation, plaque formation, and endothelial injury [22, 23]. In the chronic liver disease research, Guan et al. have proved that differential gene expressions exist in chronic hepatitis B (CHB) patients with dual deficiency of liver and kidney Yin ZHENG and accumulation of dampness-heat ZHENG [31]. Wang et al. have found that human leucocyte antigen-DR expression in CHB patients with excess or deficiency ZHENG may be different [32]. Weng et al. have screened for different gene expression in hepatocellular carcinoma (HCC) patients with or without liver-kidney Yin deficiency ZHENG [33]. According to the study of Guo et al., the gene expression was detected including two kinds of ZHENGs, liver-gallbladder dampness-heat ZHENG and liver-depression and spleen deficiency ZHENG, and two kinds of diseases, CHB and HBC. And it revealed the molecular feature of same TCM ZHENG for different disease and different TCM ZHENGs for same disease [34]. In the chronic stomach disease research, Yang et al. have observed that the metabolic levels of energy and substance are obviously reduced in spleen deficiency ZHENG, including lipid, protein, nucleic acid, carbohydrate, and trace element, which may be an important pathogenesis mechanism for its occurrence [53].

In addition, Li et al. have studied differential expression of T lymphocyte-related genes in chronic obstructive pulmonary patients with lung-Qi deficiency ZHENG by gene chips and found that there are 15 genes with high differential expression between lung-Qi deficiency ZHENG group and control group [79]. Luo et al. have investigated the abnormal change of immune function in patients with spleen-Qi deficiency ZHENG and explored the genomic mechanism by cDNA chip techniques and have confirmed that the genesis of spleen-Qi deficiency ZHENG has its immune associated genomic basis, and the immune functions are disordered in spleen-Qi deficiency ZHENG [80].

### 3.3. Proteomics

Proteins, the main carriers of biological activity, embody the next level of mRNA. Proteomics can be defined as the science and technologies associated with mapping, visualizing, and/or quantitating the expression of all or a majority of the proteins in living systems [81]. Accordingly, in addition to protein expression levels, proteomics aims to determine protein structure, modifications, localization, and protein-protein interactions. Mass spectrometry is a kind of versatile technology to measure endogenous protein, for example, liquid chromatography-mass spectrometry (LC-MS), two-dimensional electrophoresis (2-DE) combined with matrix-assisted laser desorption/ionization time-of-flight mass spectrometry (MALDI-TOF-MS), surface-enhanced laser desorption ionization time-of-flight mass spectrometry (SELDI-TOF-MS), and so on.

A large number of articles have been published on clinical TCM ZHENG differentiation research using proteomic technologies (Tables 1 and 2 and Supplementary Tables 1-2). In the coronary heart disease research, Zhao et al. have found that there are simultaneous existence of inflammatory reaction and metabolic disturbance in blood-stasis ZHENG [24, 26]. Li et al. have observed that integrin alpha-b and actin-cytoplasmic 2 are the possible marker proteins for blood-stasis ZHENG [25]. In the chronic liver disease research, Liu et al. have observed that immunoglobulin J-chains protein may serve as a novel potential biomarker for diagnosis of five kinds of different TCM ZHENGs in CHB patients [35]. By SELDI-TOF-MS technology, Song et al. established the diagnosis models of excess ZHENG and deficiency ZHENG in CHB patients [36]. And by MALDI-TOF-MS technology, Zhou et al. established the diagnosis models of spleen-Qi asthenia ZHENG, liver-kidney Yin deficiency ZHENG, and blood-stasis ZHENG in HBC patients [37]. In the hypertension research, Chu et al. have discovered the differential expressed proteins which may be the material foundation of liver-gallbladder dampness-heat ZHENG [41] and abundant phlegm-dampness ZHENG [42]. In addition, previous studies have also provided the evidence of ZHENG differentiation using proteomic technology in chronic kidney disease [48, 49], hyperlipidemia and atherosclerosis [51, 52], and chronic stomach disease [54].

Furthermore, Liu et al. have established 2-DE profiles in myasthenia gravis patients with spleen-kidney deficiency ZHENG and identified 8 significant differential proteins by MALDI-TOF-MS [82]. Lai and Fan have examined the protein fingerprint in peripheral blood mononuclear cell in systemic lupus erythematosus patients with Yin-deficiency-caused internal heat ZHENG by SELDI-TOF-MS and found the significant differences of peripheral blood mononuclear cell protein expression between Yin-deficiency-caused internal heat ZHENG group and healthy group [83].

### 3.4. Metabolomics

Another omics technology applied in systems biology research is metabolomics, involving the study of endogenous metabolites. Metabolomics aims to study the global metabolite profiles in living organisms and explore the interactions of living organisms within their surrounding environment [8, 84]. Experimental approaches in metabolomics often involve gas chromatography-mass spectrometry (GC-MS), liquid chromatography-mass spectrometry (LC-MS), nuclear magnetic resonance (NMR), and so on.

Although metabolomics started later than other omics technologies, the international and domestic scholars have also achieved several great successes in TCM ZHENG differentiation research (Tables 1 and 2 and Supplementary Tables 1-2). In the coronary heart disease research, previous research results showed that the changes of arachidonic acid, citric acid, proline, and so on may be the metabolites features of blood-stasis ZHENG [27, 28]. In the primary liver cancer research, Chen et al. have found that multiple metabolic pathways such as amino acid metabolism, lipid metabolism, glycometabolism, and energy metabolism are unbalanced or weak in Yang deficiency ZHENG [38]. In the hypertension research, blood proteins [43, 44] and urine proteins [45] were examined in hyperactivity of liver-Yang ZHENG, Yin deficiency and Yang hyperactivity ZHENG, dual deficiency of Yin and Yang ZHENG, and so on. And some significant different metabolites were identified and suggested that metabolomic approach might be a powerful tool for exploring the scientific essence of the TCM theory. In the diabetes mellitus research, several researchers have detected and classified metabolism profiles and observed significantly differential metabolites or metabolic pathways in the plasma or urine of excess and deficiency ZHENGs [56], Qi-deficiency ZHENG and Qi-Yin deficiency ZHENG [57, 58], Qi-Yin deficiency with dampness ZHENG and Qi-Yin deficiency with stagnation ZHENG [59], and Yang deficiency ZHENG and non-Yang deficiency ZHENG [60].

In addition, Li et al. have carried out a urinary metabolomic study using GC-MS in combination with multivariate statistics to differentiate two kinds of TCM ZHENGs in osteoarthritis. The results showed distinct metabolic profile separation between different TCM ZHENGs in osteoarthritis and implied that this metabolomic approach was potentially applicable as a novel strategy for TCM ZHENG differentiation and treatment of osteoarthritis [85].

The above researches revealed that the omics technologies are suitable for ZHENG global evaluation and biomarkers screening, indicated that there is indeed a molecular basis in ZHENG differentiation; there are differential expression profiles in different molecular levels, including genes, proteins, and metabolites; and there are different regulating effects on multiple moleculars, functions and pathways among ZHENGs in diseases. How to integrate systematically the information from genomics, transcriptomics, proteomics, and metabolomics, explore the molecular mechanism of TCM ZHENG theory, and find biomarkers for ZHENG differentiation will be very meaningful in future ZHENG research and clinical practice.

## 4. Applications of Omics Technologies on Clinical TCM Treatment Research Based on ZHENG Differentiation

TCM practitioners classify biomedical maladjustments into different ZHENGs, that is to say, ZHENG differentiation, also called “Bian ZHENG.” All therapeutic methods in TCM by either herbal formulae or acupuncture come from ZHENG differentiation in TCM theory for thousands of years [86]. From this point of view, ZHENG differentiation should play an important role in the effects of clinical TCM treatment. Since lack of objectivity and reproducibility, it is possible to integrate “Bian ZHENG Lun Zhi” with omics technologies leading to new scientific findings in overall medical diagnosis and treatment [15].

In modern TCM research, ZHENG-related therapeutic effect of TCM treatment has been evaluated by omics technologies, and some studies have achieved great success. By genomic approach, Xue et al. have reported that Xuefu Zhuyu Oral Liquid could improve hemorheological parameters and clinical symptoms in patients with blood-stasis ZHENG due to coronary heart disease and its relationship with human platelet antigen-3 polymorphism of membrane glycoprotein IIb [87]. By transcriptomic approach, Yu et al. have evaluated the effect of Fuzheng Huayu Capsule on patients with Qi-deficiency and blood-stasis ZHENG and verified the prediction that Fuzheng Huayu Capsule has effects on diabetes and dyslipidemia [88]. By metabolomic approach, Sun et al. have examined urine metabolites of HBC patients before and after treating with Fuzheng Huayu Tablet and found that the effect on patients with spleen deficiency with dampness encumbrance ZHENG and liver-kidney Yin deficiency ZHENG is better than that of other TCM ZHENGs [89]. Also by metabolomic approach, Sun et al. have proved that Jingqianping Granules may repair metabolic disturbance of endogenous macromolecules in premenstrual syndrome patients with liver-Qi invasion ZHENG [90]. These studies provided an evidence for TCM therapy and indicated that omics technologies are proper tools to investigate the regulatory mechanisms of ZHENG-based TCM treatment.

## 5. Discussion

In order to obtain a better therapeutic effect for human beings, ZHENG differentiation and ZHENG-based treatment are usually carried out in TCM clinical practice. The present study searched clinical TCM ZHENG differentiation research articles that used omics technologies, counted the amount of omics technologies applied in every year, summarized the most common studied diseases and TCM ZHENGs, and described the correlations between ZHENG differentiation and findings in omics studies. Moreover, this review reported the related omics technologies application of TCM ZHENG differentiation studies, and it can serve as a reference for further studies.

Though previous studies have tried to differentiate ZHENGs from different views and by different approaches, it still has some problems in the process of exploring. The first step to explore TCM ZHENG differentiation is to definitude the TCM ZHENG to study and assure the accuracy of inclusion criteria. Because of the subjectivity of TCM ZHENG differentiation, there was considerable variability across different TCM practitioners, even when the same patient was diagnosed [91, 92]. So the key challenge in TCM ZHENG differentiation research is how to standardize the diagnostic procedure for ZHENGs. In further TCM ZHENG differentiation research, the diagnostic consistency in different TCM practitioners should be paid attention to; for example, Wei et al. [93] required three practitioners reached 85% diagnosis consistency. Moreover, the computational intelligent diagnosis might be of benefit to reduce the influence of TCM practitioners' subjectivity, such as tongue diagnosis instrument and pulse diagnosis instrument [94]. Furthermore, considering the complexity of TCM ZHENG, it is advised that modern TCM ZHENG differentiation research should start with the typical ZHENG that diagnostic evidence of ZHENG such as symptoms and signs is very obvious [3].

In addition, with the characteristic of dynamic change in TCM ZHENG, a further research of the dynamic profile of ZHENG differentiation and ZHENG-based TCM treatment is needed. Particulary in ZHENG-based TCM treatment research, the dynamic profile of ZHENG should be evaluated in the process of treatment. During the experiments, the information of TCM ZHENG should be collected and recorded every once in a while in order to monitor the change of ZHENG and certify the accuracy of results. In the future, how to develop and apply kinds of high-throughput approaches, such as omics technologies may be an important topic, in dynamic ZHENG differentiation research.

The final goal of TCM ZHENG differentiation is to provide appropriate therapy for patients and get best therapeutic effect. But from previous studies screening, we found only a few articles applying omics approach on ZHENG differentiation-based treatment and mostly on animal experiments. It implied that ZHENG differentiation-based treatment research should be paid more attention to, especially on clinical practice due to the immaturity of animal ZHENG model. In further research, we should consider treatment-based ZHENG differentiation during the experiment design, in order to achieve individual therapy and get better therapeutic effects.

There are some problems in previous TCM ZHENG differentiation researches. It is derived from the complexity of TCM ZHENG, such as the standardization of the diagnostic procedure for ZHENGs, the dynamic profile of ZHENG, as mentioned above. Additionally, it is derived from the limitation of omics technologies. Though genomics could detect the whole genome and there is a fairly complete database, other omics technologies are still developing and could only detect hundreds of proteins or metabolites, which are far less than the whole proteome or metabolome. Moreover, the previous studies of ZHENG differentiation, mostly focused on quanlitative exploration, lack of quantitative evaluation, and it was hard to understand TCM ZHENG accurately and scientificly. Thus, further researches should improve omics technologies and analysis methods and pay more attention to the establishment of quantitative methods.

What is more, since the component parts of human body are structurally inseparable and functionally coordinative, TCM has paid much attention to the integrity of the whole human body and its interrelationship with nature. Meanwhile, omics technologies could explore the ZHENG mechanism or screen ZHENG biomarkers from the holistic level. This review summarized lots of clinical TCM ZHENG differentiation studies and showed that omics technologies have explored molecular basis of ZHENGs from different levels, including genes, proteins, and metabolites. Considering the integrity characteristics of TCM ZHENG, it is important to use omics approaches, kinds of technologies investigating from holistic level, to carry on TCM ZHENG researches. However, present researches are mostly applied with an omics, and there has been little progress in researches which could integrate multiomics approaches together and explain the essence of ZHENG in a whole network of human body. Therefore, how to apply bioinformatics' method to fuse information systematically from genomics, transcriptomics, proteomics, and metabolomics will also be an important issue in clinical TCM ZHENG differentiation researches.

Omics is able to provide a wide technological platform that has advantages of integrating multidimensional and multiple data for understanding the principles of TCM practice. Thus, it is believed that omics approaches could help in clarifying the essence of TCM ZHENG differentiation theory. Moreover, omics approach could assist in exploring the therapeutic mechanism of herbal medicine with the multiple components and screening the biomarkers for specific indication [3]. Therefore, omics technologies will have good application foreground on TCM ZHENG differentiation and ZHENG-based treatment.

## 6. Conclusion

With the literature analysis for the clinical research of TCM ZHENG differentiation from omics approach, we showed the trends of related articles with four omics technologies, including genomics, transcriptomics, proteomics and metabolomics, and summarized the most common studied diseases and TCM ZHENGs. In addition, we listed some typical omics applications in those common studied diseases and TCM ZHENGs. For TCM ZHENG is applied in worldwide as early, further research would pay more attention to standardizing the diagnostic procedure, revealing mechanisms from the dynamic profiles of ZHENG, establishing the quantitative methods, employing bioinformatics methods to fuse information systematically derived from multiomics technologies, discovering biomarkers for ZHENG diagnosis, and carrying out ZHENG differentiation-based treatment. Furthermore, it is also needed to develop new omics technologies and analyze methods that are appropriate for TCM ZHENG research.

## Supplementary Material

Detail methods and results of the mostly researched diseases and the most popularly studied TCM ZHENGs can be found in these supplementary tables.

## Figures and Tables

**Table 1:** The amount and percentage of the most common diseases in clinical TCM ZHENG differentiation research using omics technologies.

No.	Disease	Amount	Percentage	References
1	Coronary heart disease	24	10.43%	[18–28]
2	Chronic liver disease	15	6.52%	[29–38]
3	Hypertension	12	5.22%	[39–45]
4	Chronic kidney disease	10	4.35%	[46–49]
5	Hyperlipidemia and atherosclerosis	8	3.48%	[50–52]
6	Chronic stomach disease	8	3.48%	[53, 54]
7	Diabetes mellitus	8	3.48%	[55–60]

**Table 2:** The amount and percentage of the most common ZHENG in clinical TCM ZHENG differentiation research using omics technologies.

No.	TCM ZHENG	Amount	Percentage	References
1	Blood-stasis	26	11.30%	[18, 21–23, 25, 26, 28, 30, 35, 36, 46, 48, 50, 61–63]
2	Cold	11	4.78%	[64–70]
3	Phlegm-stasis	11	4.78%	[19, 20, 51, 52, 71]
4	Liver-kidney Yin deficiency	11	4.78%	[30–33, 35, 37, 48, 50]
5	Spleen-kidney Yang deficiency	11	4.78%	[30, 32, 35, 48, 50]
6	Dual deficiency of Qi and Yin	10	4.35%	[46, 55, 57–59]
7	Damp heat stasis in the middle-Jiao	10	4.35%	[30–32, 35, 46, 57, 61, 72]
8	Liver-depression	10	4.35%	[32, 34, 35]
9	Ascendant hyperactivity of liver-Yang	8	3.48%	[43–45, 73]
10	Phlegm-wetness	8	3.48%	[39, 40, 42, 43, 61, 74]
