# Supplementary material for: Clinical Applications of Omics Technologies on ZHENG Differentiation Research in Traditional Chinese Medicine
Source: Evid Based Complement Alternat Med. 2013 Jun 18;2013:989618. doi: 10.1155/2013/989618 (PMC3703351; doi:10.1155/2013/989618)
Supplement: Supplementary file 1 — Detail methods and results of the mostly researched diseases and the most popularly studied TCM ZHENGs can be found in these supplementary tables. [file 989618.f1.doc]

Supplementary Table 1. Applications of omics technologies on clinical TCM ZHENG differentiation research in the most diseases.

| Omics | TCM ZHENGs | Materials and Methods | Results | References |
| --- | --- | --- | --- | --- |
| [**Coronary**](app:ds:coronary) [**heart**](app:ds:heart) [**disease**](app:ds:disease) **(CHD)** | | | | |
| Genomics | Blood-stasis, non-Blood-stasis | Venous blood DNA  Examined by Taqman probe technique and gene sequencing. | No significant differences of genotypes at platelet activation related genes GPⅡb HPA-3 and GPⅠb HPA-2 could be found. | 18 |
| Genomics | Phlegm, Blood-stasis, Phlegm-stasis, and non-Phlegm-stasis | Venous blood DNA.  Examined by PCR-RFLP tech- nique. | There was a relatively close relationship between patients with ApoE ε4 allele and phlegm ZHENG. And it might be one of the main susceptible genes in CHD patients with phlegm ZHENG. | 20 |
| Transcriptomics | Blood-stasis, non-Blood-stasis | Venous blood RNA.  Examined by gene chip technique, and confirmed RT-PCR. | The relationship of inflammatory- and immune-related genes with CHD patients with blood-stasis was revealed at the level of nucleic acid. | 22 |
| Transcriptomics | Blood-stasis, non-Blood-stasis | Venous blood RNA.  Examined by gene chip technique, and confirmed by RT-PCR. | The hereditary related differential genes of blood-stasis ZHENG in CHD patients were closely associated with inflammation, plaque formation and endothelial injury. | 23 |
| Proteomics | Qi-deficiency and blood-stasis | Plasma protein.  Exzamined by label-free quanti- tative technology based on LC- MS/MS. | Qi-deficiency and blood-stasis ZHENG in CHD patients might belong to a kind of inflammatory reaction. There were simultaneous existence of myocardial injury, blood coagulation factor abnormality, lipid metabolic disorder and oxygen transport obstacle, and they influenced and interacted mutually. | 24 |
|  |  |  |  |  |
| Omics | TCM ZHENGs | Materials and Methods | Results | References |
| Proteomics | Blood-stasis | Platelet protein.  Examined by 2-DE, indentified by MALDI- TOF-MS, and confirmed by Western blot. | Integrin alpha-b and actin-cytoplasmic 2 were the possible marker proteins, and other platelet functional proteins might play crucial roles in the occurrence and development of blood-stasis ZHENG in CHD patients. | 25 |
| Proteomics | Qi-deficiency and blood-stasis, Phlegm- stasis | Plasma protein.  Examined by 2-DE and tandem mass spectrometry. | The proteomics characteristics of patients with Qi-deficiency and blood-stasis ZHENG and phlegm-stasis ZHENG might have relationships with inflammatory reaction and metabolic disturbance. | 26 |
| Metabolomics | Heart-blood stasis, non Heart-blood stasis | Plasma metabolite.  Examined by GC-MS. | Analyzed from the view of plasma metabolites, heart-blood stasis ZHENG in CHD patients was related with lipid metabolism and glycometabolism, also with the stress induced by hypoxia and agonia. | 27 |
| **Chronic liver disease** | | | | |
| Genomics | Excess, Deficiency | Venous blood DNA.  Examined by PCR-LDR technique. | The result showed that Interleukin-10-819C/T was significantly correlated with Deficiency ZHENG in HBC patients. | 29, 30 |
| Transcriptomics | Liver-kidney yin deficiency, Liver-gallb- ladder dampness-heat | Venous blood leukocyte DNA.  Examined by gene chip technique. | A total of 239 genes were significantly differentially expressed, among which 142 were up-regulated and 97 were down-regulated. | 31 |
| Transcriptomics | Liver-gallbladder dampness-heat, Liver depression and spleen deficiency | Venous blood leukocyte DNA.  Examined by gene chip technique. | The study explained the mechanism of different TCM ZHENGs for same disease and same TCM ZHENG for different diseases on the level of RNA. | 34 |
|  |  |  |  |  |
| Omics | TCM ZHENGs | Materials and Methods | Results | References |
| Proteomics | Damp heat stasis in the middle-Jiao, Liver Qi stagnation and spleen deficiency, Spleen and kidney Yang deficiency, Liver and kidney Yin deficiency, Blood stasis into collateral | Plasma protein.  Examined by MALDI-TOF-MS, and confirmed by ELISA. | In the cases of five TCM ZHENGs in CHB, immunoglobulin J-chains and C-reactive protein were up-regulated, while haptoglobin, retinol binding protein and vitronectin were down-regulated. Moreover, the up-regulated immunoglobulin J-chains level in plasma was related with the different TCM ZHENGs in CHB patients. | 35 |
| Proteomics | Excess, Deficiency | Serum protein.  Examined by SELDI-TOF-MS. | The study provied the possibility of TCM ZHENG differentiation in CHB patients using a universally acceptable scientific approach. | 36 |
| Metabolomics | Yang-deficiency, non-Yang-deficiency | Serum metabolite.  Examined by NMR. | The down-regulated metabolites might be the distinctive metabolic variations of Yang deficiency ZHENG. And these metabolites might be potential biomarkers for diagnosis. | 38 |
| [**Hypertension**](app:ds:hypertension) | | | | |
| Genomics | Liver-fire exuberant, Dual deficiency of Yin and Yang | Venous blood DNA.  Examined by PCR-RFLP tech- nique. | Gene mutation of AGT M235T might be associated with the genesis and development of hypertension, and TCM ZHENG had its own intrinsic molecular biological foundation. | 40 |
| Proteomics | Liver-gallbladder dampness-heat, non-Liver- gallbladder dampness-heat | Serum protein.  Examined by MALDI-TOF-MS. | The differently expressed proteins were the material foundation of liver-gallbladder dampness-heat ZHENG. Decision model could offer a tool for TCM ZHENG differentiation more objectively and accurately. | 41 |
| Proteomics | Abundant phlegm-dampness, non-Abundant phlegm-dampness | Serum Protein.  Examined by MALDI-TOF-MS. | There were 102 differentially expressed protein peaks between abundant phlegm-dampness ZHENG and control group. Four protein peaks were screened out which could induce abundant phlegm-dampness ZHENG. | 42 |
| Omics | TCM ZHENGs | Materials and Methods | Results | References |
| Metabolomics | Hyperactivity of liver Yang, Abundant phlegm-dampness, Yin deficiency and Yang hyperactivity | Serum metabolite.  Examined by GC-MS. | Mahlanobis distance analysis contributed not only to a fine differentiation, but also to a clear exhibition of the progression, of three TCM ZHENGs. | 43 |
| Metabolomics | Hyperactivity of liver Yang, Dual deficiency of Yin and Yang | Plasma metabolite.  Examined by LC-TOF-MS. | Estrodiol, leucotriene, ceramide etc. increased more in hyperactivity of liver Yang ZHENG, while triglyceride and diacylglycerol increased more significantly in dual deficiency of Yin and Yang ZHENG. | 44 |
| Metabolomics | Hyperactivity of liver Yang | Urine protein.  Examined by LC-TOF-MS. | This study found out some small molecular metabolic markers from the microscopic field, which indicated metabolomics might help to probing the biological nature of TCM ZHENG. | 45 |
| **Chronic kidney disease** | | | | |
| Genomics | Dual deficiency of Qi and Yin, Liver and kidney Yin deficiency | Peripheral blood mononuclear cells DNA.  Examined by gene sequencing. | A267G in 5’-untranslated region within exonal of megsin gene might be one of the substantial genetic basis for differentiating the two TCM ZHENGs in primary immunoglobulin A nephropathy patients. | 47 |
| Proteomics | Kidney Yin deficiency, Kidney Yang deficiency | Plasma protein.  Examined by 2-DE and MALDI- TOF-MS. | Compared with control group and chronic glomerulonephritis kidney Yang deficiency ZHENG group, there were 80 highly expressed protein spots and 42 lowly expressed protein spots in the 2-DE images of kidney Yin deficiency ZHENG group. | 49 |

Supplementary Table 2. Applications of omics technologies on most clinical TCM ZHENG differentiation researches.

| Omics | Diseases / Sub-health | Other TCM ZHENGs | Materials and Methods | Results | References |
| --- | --- | --- | --- | --- | --- |
| **Blood-stasis** | | | | | |
| Geomics | Sub-health | Yin-deficiency, Qi-deficiency, Wetness-heat, Yang-deficiency, Phlegm-wetness | Venous blood DNA.  Examined by PCR-sequencing-based typing method. | An association between TCM constitution and HLA polymorphism was indeed found to exist. | 61 |
| Transcriptomics | CHD | None | Venous blood RNA.  Examined by oligonucleotide microarray technique, and confirmed by RT-PCR. | Inflammatory- and immune-related genes acted as a pivot in blood stasis ZHENG gene expression profiles, which might explain the function of inflammation and immune response in the occurrence and progress of blood stasis ZHENG. | 62 |
| Proteomics | Rheumatoid arthritis (RA) | Non-Blood-stasis | Plasma protein.  Examined by SELDI-TOF-MS, and analyzed by hierarchical clustering and decision tree. | 266 protein peaks were found, whose expression was significantly different between blood-stasis and non-blood-stasis. | 63 |
| Metabolomics | CHD | None | Urine metabolite.  Examined by NMR, and analyzed by pattern recognition technique. | The changes of some metabolites might be associated with the metabolic characteristics in the blood-stasis patients, and the study provided a new idea for diagnosis, treatment and TCM ZHENG research. | 28 |
|  |  |  |  |  |  |
| Omics | Diseases / Sub-health | Other TCM ZHENGs | Materials and Methods | Results | References |
| **Cold** | | | | | |
| Transcriptomics | None | None | Periheral CD^+^_4_ RNA.  Examined by gene chip technique. | NEI interaction not only revealed the mechanism of classical TCM theory on ZHENG but also enriched current researches on complex diseases as well as systems biology. | 65 |
| Transcriptomics | RA | Heat | Periheral CD^+^_4_ RNA.  Examined by gene chip technique. | The results suggested TCM ZHENG differentiation had its own basis of gene expression profile. | 67 |
| Transciptomics and Metabolomics | RA | Heat | Periheral CD^+^_4_ RNA and plasma metabolite.  Examined by gene chip technique and UPLC-TOF-MS or GC-MS. | The transcriptomics and metabolomics analysis showed statistically different gene expression and metabolite profiles between Cold and Heat ZHENGs, and molecular differences between TCM ZHENG groups were found. | 68, 69 |
| Metabolomics | RA | Heat | Plasma metabolite.  Examined by UPLC-TOF-MS, and analyzed by pattern recognition technique. | The subtypes of RA diagnosed based on modern medicine (active stage and stable stage) and traditional Chinese medicine (Cold ZHENG and Heat ZHENG) were separated clearly by pattern recognition. | 70 |
|  |  |  |  |  |  |
| Omics | Diseases / Sub-health | Other TCM ZHENGs | Materials and Methods | Results | References |
| **Phlegm-stasis** | | | | | |
| Genomics | CHD | Non-Phlegm-stasis | Venous blood DNA.  Examined by gene sequencing. | Apo Eε4 allele was a susceptible allele to CHD, which was closely related to phlegm-stasis ZHENG. It suggested that it might be one of important susceptible alleles for phlegm-stasis ZHENG. | 19 |
| Proteomics | Hyperlipidemia and Athrosclerosis | Phlegm, Blood-stasis , non-Phlegm-stasis | Plasma protein  Examined by two-dimensional polyacrylamide gel electrophoresis and mass spectrometry. | Phlegm-stasis patients could be separated from non-phlegm-stasis ones by fibrinogen βchain related with the deficiency of kidney-Qi, and apolipoprotein AⅠ precursor related with both the deficiency of kidney-Qi and heart-Qi. | 51, 52 |
| Metabolomics | Tumor | Non-Phlegm-stasis | Plasma metabolite.  Examined by NMR, and analyzed by pattern recognition technique. | Compared with tumor patients of non-phlegm-stasis ZHENG, patients of phlegm- stasis ZHENG had obvious lower plasma contents of leucine, alanine, citrulline and so on, higher plasma contents of acetone, unsaturated fatty acid, glycoprotein and so on. | 71 |
| **Liver-kidney Yin deficiency** | | | | | |
| Genomics | Primary hyperlipemia | Spleen-kidney Yang deficiency, Phlegm-stagnation, Qi-stagnation caused blood stasis | Venous blood DNA.  Examined by gene sequencing. | The results indicated that polymorphism of ApoE gene was related in a certain degree to TCM ZHENG of primary hyperlipemia. | 50 |
|  |  |  |  |  |  |
| Omics | Diseases / Sub-health | Other TCM ZHENGs | Materials and Methods | Results | References |
| Transcriptomics | HCC | Non-Liver-kidney Yin deficiency | Peripheral blood mononuclear cells RNA.  Examined by gene chip technique, and confirmed by RT-PCR and Western blot. | The results confirmed the hypothesis on the essence of TCM ZHENG, that was to say, multi-gene changed from normal to morbid state to on the level of mRNA and protein | 33 |
| Proteomics | Chronic renal failure | Spleen-kidney Qi deficiency, Spleen-kidney Yang deficiency, Dual deficiency of Yin and Yang | Urine protein.  Examined by SELDI-TOF-MS. | Urine protein biomarkers could reflect different biological characteristics of chronic renal failure patients of different TCM ZHENGs to some extent. | 48 |
